# Horticultural activities can achieve the same affect improvement effect of green exercise: A randomized field controlled trial

**DOI:** 10.3389/fpsyg.2022.989919

**Published:** 2022-11-16

**Authors:** Meng Tao, Li Lu, Jingchuan Gao, Xiaolong He

**Affiliations:** Department of Physical Education and Health Science, Zhejiang Normal University, Zhejiang, China

**Keywords:** horticultural activities, green exercise, affect, heart rate variability, health

## Abstract

**Objectives:**

With the deepening of non-drug intervention research on human mental health, more and more attention has been paid to the benefits of horticultural activities and green exercise on physical and psychological health. This study compared the affect improvement between horticultural activities with the same intensity and green exercise and that with or without green plants to verify the value of horticultural activities and green exercise in improving human affect and the importance of green plants.

**Methods:**

A total of 160 subjects aged 18–26 years (average age 22.5 years) were recruited and randomly divided into a control group, a horticultural activity group with green plants, a horticultural activity group without green plants, and a green exercise group. Demographics, sociological variables, and daily physical activity levels were investigated. Green space at Zhejiang Normal University was selected as the test site. After finishing the preparation work, the subjects sat quietly for 8 min before the pre-test. The horticultural group completed 20 min of horticultural activities {8 min of digging [40%*HRR(heart rate reserve) + RHR(resting heart rate)] + 8 min of transplantation [(50%*HRR + RHR) + 4 min of watering (30%*HRR + RHR)]}. The group returned to a calm state (no less than 20 min) for the post-test. The green exercise group completed a 20-min power bike ride. The activity intensity and activity time of the green exercise group were determined according to the activity intensity and time of the horticultural group. Dependent variables were collected, including blood pressure, positive/negative affects, heart rate variability (RMSSD, SDNN, and LF/HF), and controlled covariate environmental parameters (field temperature, humidity, and noise).

**Results:**

(1) A significant difference was observed in the improvement effect except for negative affect between the green horticultural activity group and the green exercise group (*F* = 3.310; *ɳp^2^* = 0.046; *p* = 0.037). No significant difference was observed in other affect indicators. (2) In the same pattern of with and without green plant horticultural activity group, the green plant horticultural activity group had a better effect on the improvement of affect, and the two groups had a better negative affect (*F* = 3.310; *ɳp^2^* = 0.046; *p* = 0.037), SDNN index of heart rate variability(*F* = 1.035; *ɳp^2^* = 0.015; *p* = 0.039), and RMSSD index (*F* = 2.225; *ɳp^2^* = 0.032; *p* = 0.014), and no significant difference was observed in the improvement effect of other affect indicators between the two groups.

**Conclusions:**

Having green horticulture can give the same intensity as green exercise and affect improvement. Findings suggest that people can choose green exercise or horticultural activities according to their preferences and physical characteristics in the two physical activities. Under the same pattern of horticultural activities, green plants are the key factor in improving the affect of horticultural activities. Choosing suitable plant types in horticultural activities is positively significant in enhancing affect.

## Introduction

World Health Organization (WHO) statistics show that mental health disorders have gradually become one of the leading chronic non-communicable diseases threatening global human health (Bo et al., 2017). In recent years, the international effort against human mental health problems has extended to non-drug interventions, such as physical activity and environmental research of psychological therapy; physical activity interventions have positive effects on metabolic syndrome-related diseases, can improve mental health, stimulate positive affect, improve the sense of social value, and promote interpersonal communication (Amireault et al., 2017; Kadariya et al., 2019). Compared with single physical activity, many empirical studies have found that interventions that can reasonably combine physical activity and green plants can play a better role in the effect of physical and mental health interventions (Clatworthy et al., 2013; Rowinski et al., 2015). As one of the effective ways to combine physical activity with green plants, horticultural activity can provide people with physical activity and increase the opportunity for people to have close contact with natural elements. Approximately 27 million people in the United Kingdom (40% of the population), 117 million people in the United States (35% of the population), and 32 million people in Japan (25% of the population) use horticultural as a way of promoting physical and mental health daily (Wood et al., 2016).Overall, horticulture is suitable for all groups and effectively improves physical exercise, cognitive function, life satisfaction, and quality of life (Brown et al., 2004; Nicklett et al., 2016).Through physical activity, interactions with nature or greenery can enhance positive associations between an individual’s mental, social, and physical health, which may benefit from interacting with nature. At the same time, in addition to environmental psychotherapy, many cross-sectional and longitudinal studies have confirmed that long-term exposure to a good environment, such as lawns, shrubs, and trees composed of green landscape, has positive effects on mental health conditions, such as depression, anxiety, and stress (Pretty et al., 2005; Gascon et al., 2015; Olafsdottir et al., 2017). Many controlled trials have found that even one total exposure to the green landscape affects mental health (Alcock et al., 2014; Rogerson et al., 2016a).Numerous studies have shown that physical activity in the outdoor environment is better than static relaxation in the green space to improve human mental health and is better than indoor activity with the same activity mode and intensity. The concept of green exercise and related research has been rapidly developed (Yi et al., 2016; Rautio et al., 2018).

Searching of the literature found that horticultural activities positively improve human affect. However, in many studies, the comparative experiments on horticultural activities and green exercise did not seriously consider the intensity of the horticultural activities. Many studies showed that horticultural activities also involve considerable high-intensity movement. This study can provide adequate data support for horticultural activities and green exercise to improve human affect through randomized controlled experiments. Moreover, by comparing the differences between the effect of green plants and no green plants on the improvement of human affect under the same horticultural activity mode, this study confirms that green plants are the critical elements improving the effectiveness of horticultural activities, which can provide a basis for future research in this field. This study recommends variable adjustment and individualized intervention plans for groups with different characteristics.

## Materials and methods

### Participants

The present study referred to the previous research design of green exercise and horticultural activities to improve the subjects’ affect (Gidlow et al., 2016; Fraser et al., 2019). The international software G*Power 3.1 was used to estimate the number of issues. In G*Power, the f-test, repeated measures ANOVA, main effect, and interaction effect were used as the test method. In addition, a previous short-time intervention control experiment design was referenced. The related parameters were set as follows: the α significance level was 0.05, effect size (ES) was 0.25, and power was 0.80. Moreover, the number of the test groups was four, and the measurement time was two. The final G*Power output was 36 participants in each group. Before the start of the experiment, the sample loss percentage was fully considered. The target participants were college students, and 160 individuals were recruited according to the requirements of the questionnaire. The age range of the participants was 18 to 26 years. The students were grouped randomly into a control group (CG), a horticultural activity group with green plants (HA [a]), a horticultural activity group without green plants (HA[b]), and a green exercise (GE) group. In the beginning stage, five participants did not complete the experiment owing to the abnormal signal reception of the heart rate belt, and three participants had missing data after the data exporting; thus, only 152 participants completed the investigation successfully.

**Figure 1:**
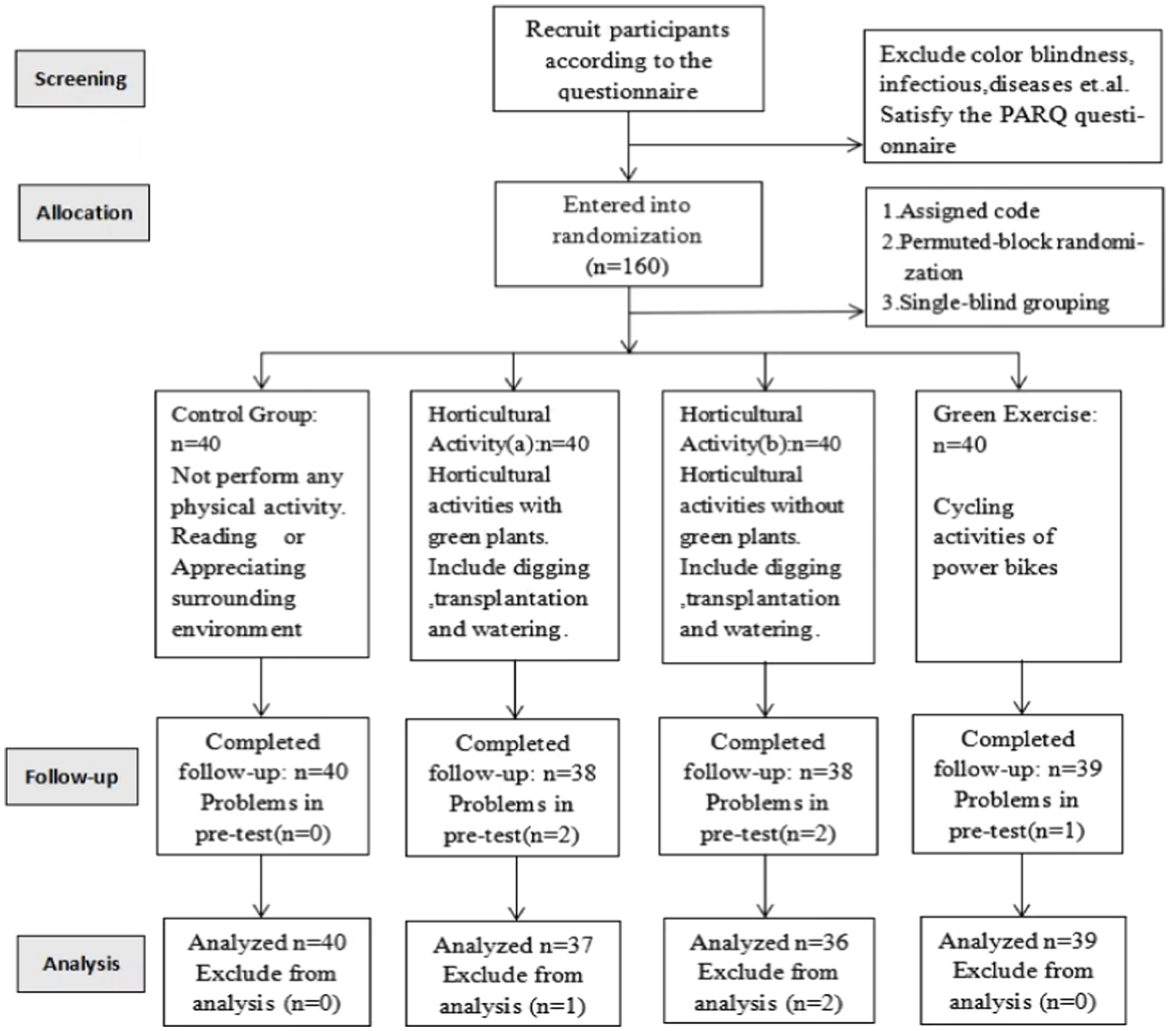
Consort chart showing the recruitment, random allocation, follow-up, and analysis of the participants.

An independent team member who was not involved in any other part of the project was responsible for the random allocation. Each recruited participant was assigned a code, and permuted-block randomization was employed to designate the participants by regulation to the experimental or control conditions after the baseline data collection. The team member responsible for the randomization was blinded against the participants’ profile, and the data collectors were blinded against the participants’ grouping throughout the study period. The flowchart of the test assignment is shown in Figure 1.

### Questionnaire survey

Before the experiment, the recruited participants were investigated using a questionnaire to understand the situation for each issue. The questionnaire mainly collected the participants’ (a) basic information, such as gender, age, education, family structure, and economic situation, and (b) daily physical activity, such as type, frequency, and intensity. The reliability and validity of the questionnaire used in this study were tested effectively, and the questionnaire was used in previous studies. Therefore, the questionnaire on the demographic and sociological variables in this study was rigorous and valid. At the same time, some covariates in this study were based on exploratory properties, such as environmental noise, air humidity, and temperature.

All the recruited subjects volunteered to participate in this study. The subjects first read the informed consent of the experiment and agreed to procedures that would be applied in the study. The ethics committee approved this academic research study of Zhejiang Normal University (Project No.: zsdd2021011). After obtaining the subjects’ permission, they filled in the scales of population sociology variables and other variables. When the subjects were recruited to participate in the study, they would be told not to drink alcohol for 5 days before the test and to avoid coffee, tobacco, and some drugs for 2 days.

### Place and time of the experiment

**Figure 2:**
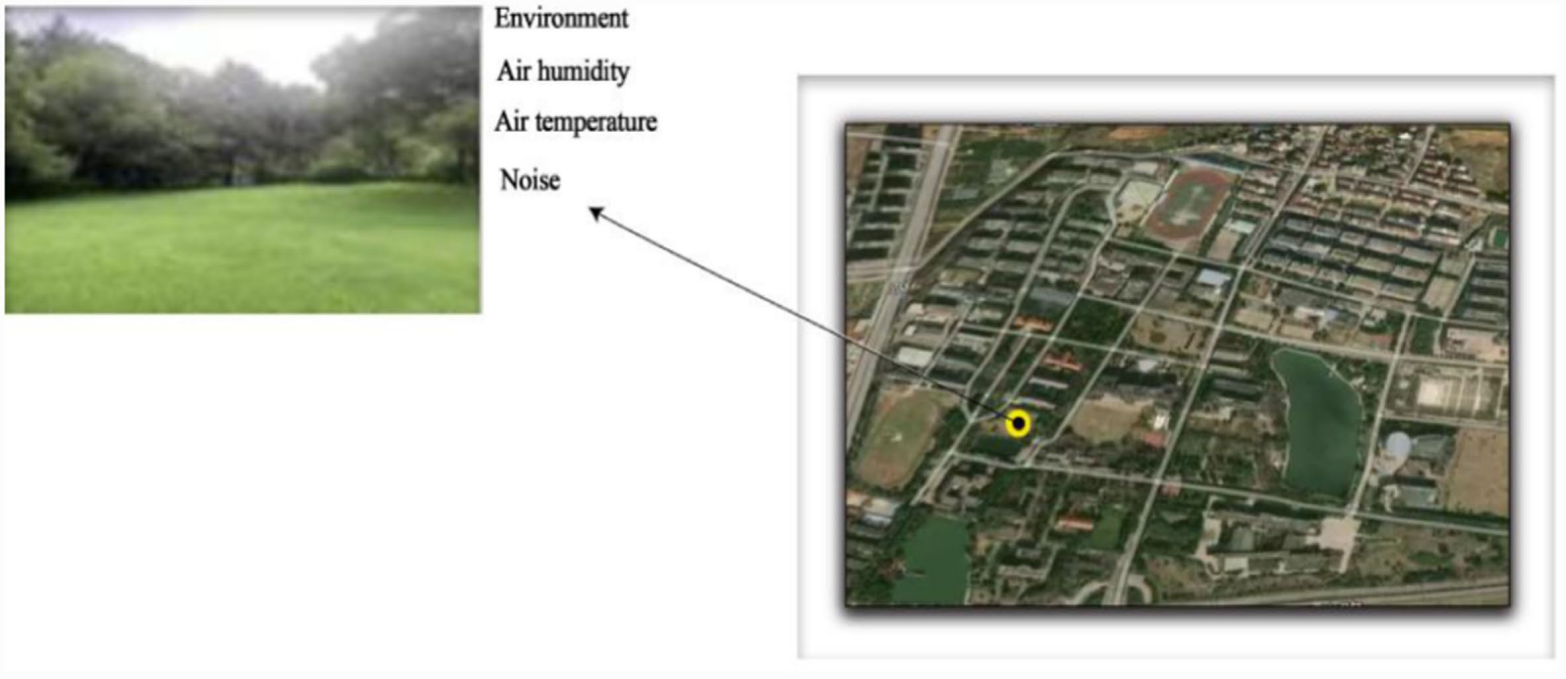
Satellite image and real image of the experimental test site.

The test was carried out on the campus of Zhejiang Normal University in Jinhua, Zhejiang Province. Figures 2 is the satellite and real pictures of the test site. Referring to the previous study design, the test time of this study was mainly carried out from April to June 2021. At this time, the environment and climate in the Wucheng area of Jinhua City were generally suitable, and the air temperature and humidity were also comfortable. The influence of high or low temperatures in summer and winter on the subjects’ affective state and the experimental test results could be avoided in these two periods.

The specific test time was arranged in the afternoon from 15:00 to 16:30 and from 16:30 to 18:00, Monday to Friday. Thus, the test can avoid the possible intense solar irradiation before 15:00 and the influence of the gradual dimming of light after 18:00 during the collection of the dependent variables of the subject to eliminate the interference of the experimental results. The specific test time for each subject was approximately 50 min. A relatively sufficient test time must be ensured given that preparing before and tidying up after the test take some time. The test weather must be a relaxing sunny day, and the ambient temperature of the outdoor test was approximately 25°C to 30°C. Air temperature and humidity were suitable. The test was not carried out on rainy days or in particular weather conditions.

### Experimental process

**Figure 3:**
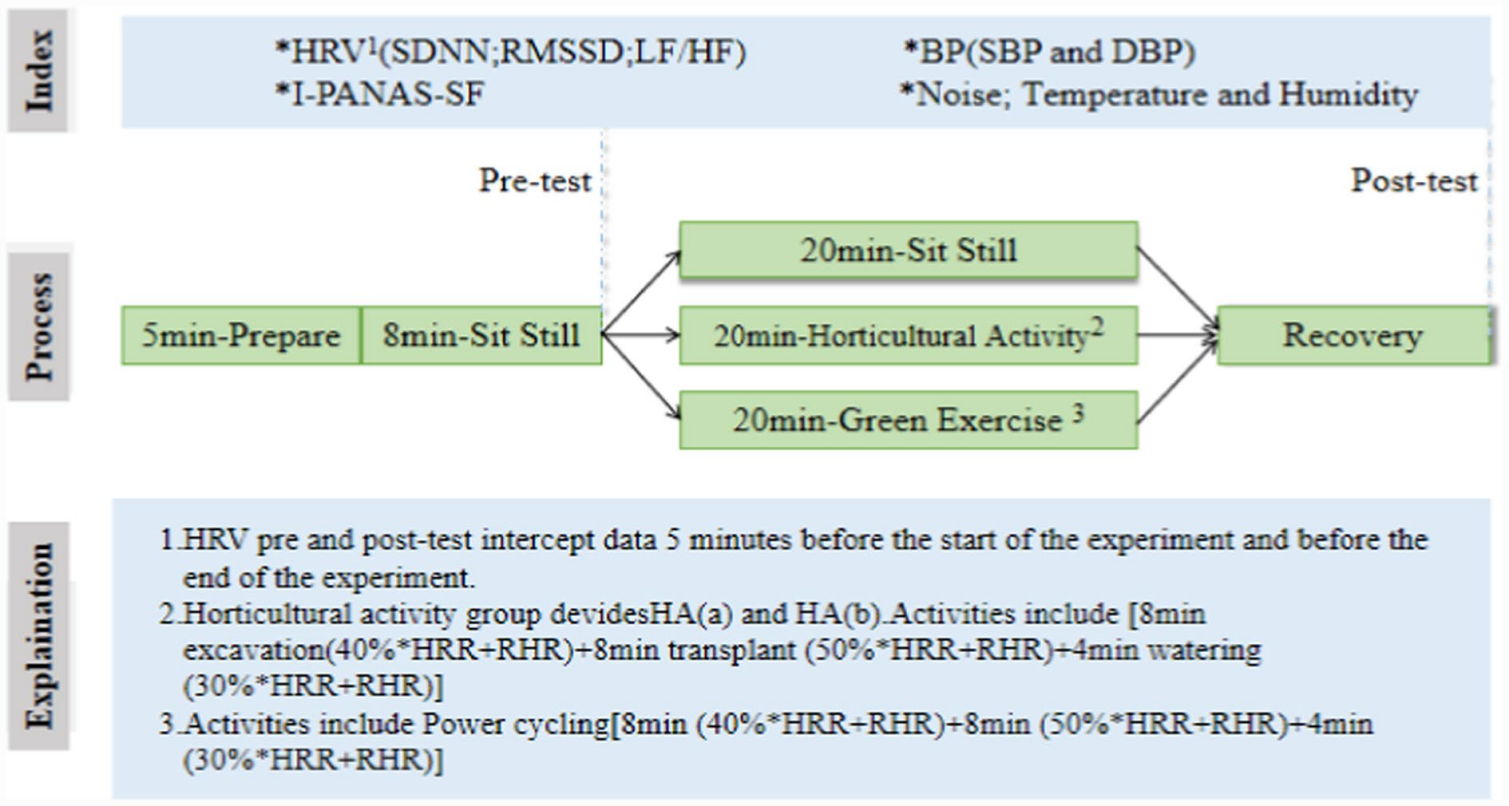
Experiment specific flow chart.

The physical intensity of green exercise and horticultural activities may vary slightly depending on geographical location and external factors, such as climate, altitude, weather, environment, and soil type. Therefore, measures for green exercise and horticultural activities with the same intensity must be adequate, and controlled experimental research must be carried out in the same geographical environment. Each practice test was divided into four stages: preparation, pre-test, intervention, and post-test. The specific experimental process is shown in Figure 3.

1. **Preparation stage:** The staff prepared relevant experimental equipment for the test. After the subjects arrived at the test site, the team introduced the practical test instructions. After reading the ethical approval document, the issues filled in the informed consent for this experiment and started the formal test after obtaining the informed consent of the problems. The preparation period lasted approximately 5 min.
2. **Pre-test stage:** Before activity intervention, the subjects’ individual demographic and social variables, daily physical activity level, and other information were collected. The dependent variables were collected in the following order: ① The subjects’ systolic and diastolic blood pressure was measured by Omron electronic sphygmomanometer. ② A First Beat wearable wireless physiological device was used to collect heart rate variability (HRV) data 5 min before the experiment. ③ The issues were noted in real-time in the “positive affect scale” and “negative affect scale.” The previous test period was approximately 8 min.
3. **Intervention stage:** The control group (CG) consisted of the participants who were not included in any physical activity intervention after arriving at the experiment site. To prevent the participants from being bored, they were not required to remain sedentary for a long period of time. During this stage, they could choose to read according to their preference or appreciate the surrounding environment freely to relax, which lasted approximately 20 min.

The horticultural activity groups included those with green plants (HA[a]) and without green plants (HA[b]). Before the experiment on horticultural activities, the staff conducted several pre-experiments on horticultural activities. On the premise of the intensity of pre-experimental horticultural activities and experimental data, the target rate of complete and continuous horticultural activities was found to increase first and then decrease. Based on the analysis of the pre-experimental data, the intervention contents of horticultural activities mainly included three endless physical activities: digging, transplanting, and watering. The specific content is as follows: ① Excavation: The subject needs to excavate the soil trough that has been filled with soil to prepare for the planting of green plants. The excavated soil was poured into the empty soil trough prepared in advance for the following physical activity of transplanting. At this stage, the subjects’ target rate of activity intensity was maintained at approximately 40%*HRR + RHR, lasting approximately 8 min. ②Transplanting: The issues transplanted the green plants into the empty soil trough after excavation. The target rate of activity intensity of the problems was maintained at approximately 50%*HRR + RHR, lasting for approximately 8 min. ③ Watering: the green plants were watered with a kettle filled with water. The target rate of activity intensity was maintained at 30%*HRR + RHR, and the duration was approximately 4 min. In the process of horticultural intervention, when the subjects’ heart rate began to deviate from the target heart rate, the volume of soil, soil tank volume, and kettle volume in the soil tank were controlled to adjust the target heart rate back to the target heart rate. The target heart rate range was ±10 times/min, and the real-time heart rate deviated from the target heart rate ± 5 times/min without warning. When the time was ±5 to ±10 times/h, the staff reminded the subjects to make a moderate adjustment to make the heart rate return to the target heart rate, which lasted approximately 20 min.

**Figure 4:**
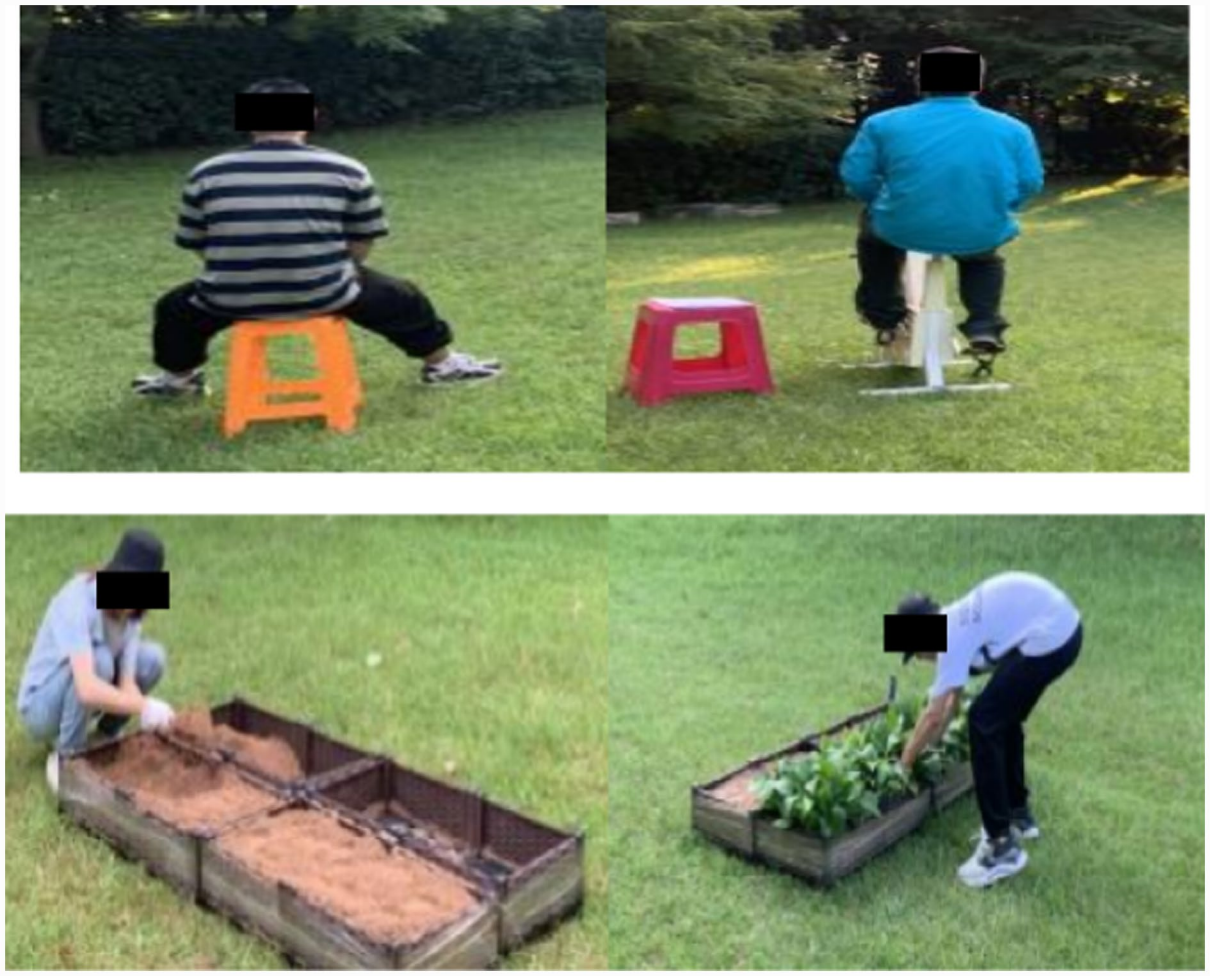
The scene photos of the sitting control group, the green exercise group, and the horticultural activity group.

In the green exercise group (GE), the green exercise group’s activity intensity and activity time should be consistent with that of the horticultural activity group under the horticultural activity group’s bullseye rate of activity intensity. Participants were arranged to ride a power bicycle in Sweden (MONARK) for 20 min. The specific activity process was as follows: ① The subjects carried out the power bicycle cycling activity under the activity intensity of maintaining the target rate of approximately 40%*HRR + RHR, and the duration was approximately 8 min (consistent with the digging stage of horticultural activity). ② The subjects carried out power cycling activities at the activity intensity of 50%*HRR + RHR, lasting approximately 8 min (consistent with the planting stage of horticultural activities). ③ The subjects carried out power cycling activities at the activity intensity of maintaining the target rate of approximately 30%*HRR + RHR, and the duration was approximately 4 min (consistent with the watering stage of horticultural activities). This stage took approximately 20 min. Shown in Figure 4, photos of each group during the experiment.

4. **Post-test stage:** After the physical activity intervention, the heart rate band could be removed only when the subjects’ heart rate was restored to the heart rate in a calmer state than before the experiment. The recovery time of the issues was no less than 20 min. Compared with the quiet heart rate of the subjects before the investigation, the subjects’ heart rate could be maintained for 30 s or more, and then the post-test was started. In the post-measurement stage, the collection sequence of dependent variables was consistent with that in the pre-measurement location: ① The systolic and diastolic blood pressure were collected by Omron electronic sphygmomanometer. ② A First Beat wearable wireless physiological device was used to collect HRV data 5 min before the end of the experiment. ③ The subjects noted in real-time the “positive affect scale” and “negative affect scale.” This stage lasted approximately 20 min.

After completing the physical activity intervention, the staff provided 500 ml of portable pure water to the subjects, who could choose whether to drink pure potable water or not according to their actual situation and needs. The subjects must not drink pure water during the whole recovery stage after drinking water for the first time to avoid unnecessary interference with HRV and other indicators during the recovery stage. Furthermore, when the staff collects dependent variable indicators in the pre-test and post-test phases, they also need to manage environmental noise, air temperature, and air humidity in the pre-test and post-test stages, which will be included in the subsequent model analysis.

### Dependent variable indicators

#### Heart rate variability

In the green exercise and horticultural activities-related control experiments, LF/HF is often used as the frequency domain analysis index, mainly reflecting the balance between the sympathetic nerve and vagus nerve or the modulation degree of the sympathetic nerve. RMSSD and SDNN were primarily used as comprehensive indicators of affect improvement in short-term intervention control experiments (Rhie et al., 2017; Sin-Ae et al., 2017). Thus, this experimental study mainly collected LF/HF, RMSSD, and SDNN indexes for processing and analysis.

For the HRV index collection, this study selected the First Beat Sports wireless ECG module of physiological data collection system equipment. The equipment system can perform real-time detection and agile changes to capture subjects’ heart rate, and the background can be heart rate changes of a signal automatically converted into time domain and frequency domain data for record-keeping. The ECG module device of the First Beat Sports wireless physiological data collection system has been used in many controlled experiments on green exercise and horticultural activities. The device system’s real-time accuracy and reliability of data recorded have been verified in many controlled experiments (Corrales et al., 2012; Beauchaine and Thayer, 2015).The HRV data were imported into an Excel sheet to process the calculations, and the exported data were divided into a time-domain analysis table and frequency-domain analysis table. The calculations used were all corrected RR values, and the specific calculations for the root mean square of the difference between adjacent RR intervals (RMSSD), standard deviation of the continuous normal RR period (SDNN), and ratio of LF to HF (LF/HF) are described below.

### Time-domain indicators

1. SDNN: This indicator is calculated in Excel by applying the SD formula to the RR values. For example, the data in cell A1:A4 = STDEVP(A1:A4).
2. RMSSD: The Excel calculation for this indicator is as follows: (a) copy the RR interval value column, stagger it before and after the “-” front of the value, then drop down “+,” resulting in the adjacent interval difference, then (b) calculate the RMS. For example, the data in cell A1:A4 = SQRT(SUMSQ(A1:A4)/COUNTA(A1:A4)), where SQRT calculates the square root of an arithmetic number, SUMSQ calculates the sum of the squares of several numbers, and COUNTA calculates the total number of numbers. Frequency-domain indicators.
3. LF/HF: This indicator is calculated in Excel as follows: = LF/HF, then, drop down “+” to get the average value of LF/HF for the time period.

#### Blood pressure

BP includes systolic BP (SBP) and diastolic BP (DBP). A decrease in BP can reflect a reduction in the negative affect of an individual to a certain extent. Conversely, BP improvement can reflect the enhancement of an individual’s mental health status (Jules, 2004; Zijlema et al., 2018; Quan M et al., 2021). An Omron upper arm electronic sphygmomanometer (HEM-7121) was used to measure SBP and DBP. The accuracy and reliability of this brand in measuring BP indicators were confirmed in many studies. Before the start of the experiment, the health professionals were asked to calibrate the sphygmomanometer, including the size of the cuff’s balloon, accuracy of the pressure sensor, and accuracy of the algorithm. During the electronic sphygmomanometer reading, the subjects kept breathing calmly and avoided limb movement. At the end of one measurement, the data were recorded after the reading stabilizes. After 1–2 min, the above steps were repeated to measure again. The average value of the two measurements was taken. The arm should be the same when measuring the subject’s BP to avoid the difference in BP measurement caused by different arms.

#### Positive/negative effects

The affect index can record and evaluate the mental health status of subjects in real-time. As for the real-time affective level of the issues, the international general positive/negative affect scale (I-PANAS-SF short volume version) was used to evaluate the real-time affective status of the subjects. At present, the universality and reliability of the scale have been confirmed in many controlled experiments (Thompson and E, 2007; Liu et al., 2012). The short volume version of the positive/negative affect scale included five positive and five negative questions. Participants were asked to rate positive and negative questions based on their real-time affective status in the pre-test and post-test phases. Finally, the total positive and negative scores were calculated to reflect the subjects’ affective states in the pre-test and post-test stages.

### Covariable indicators

For outdoor field tests, weather conditions inevitably change. Air temperature, humidity, and noise pollution also differ in different seasons and, at other times, inevitably cause interference to the test subjects. Therefore, real-time monitoring of air temperature, humidity, and noise changes is usually conducted in many outdoor experimental tests (Bringslimark, 2007; Kim et al., 2021). The Simma AS817 instrument monitored air temperature and humidity in this experiment. The Dongmei JHT-80A instrument was used for real-time tracking field noise decibel.

At the two time points of pre-test and post-test dependent variable collection, the staff used the above equipment to record the environmental parameters of the outdoor field test. Considering that the impact of environmental parameters in field outdoor testing on the variation difference of dependent variables of subjects is often not a single time node but may be caused by continuous accumulation to a specific period, the average values of environmental parameters in the pre-test and post-test stages were taken. The average temperature, humidity, and noise values were incorporated into the statistical model as covariables for analysis.

### Statistical methods

After the data collection was completed and the invalid data were eliminated, the data were entered into Excel 2010 and saved. Next, the Excel data were imported into SPSS 23.0 for processing, including several procedures. (1) Descriptive statistical analysis was conducted on the dependent variable indicators in the pretest and posttest stages in the form of mean ± SD. Based on the *f*-value, value of p, and ES ɳp^2^ of the multivariate ANOVA, the improvement effects of the dependent variable indicators on each group in the pretest and posttest stages were compared, and gender, age, education level, mean temperature, mean humidity, and mean noise were included as covariates in the statistical model to control. (2) The f-value, value of p, and ES ɳp^2^ of the repeated measures ANOVA were used in the general linear models to test the relationship between the different groups (including HA [a] and GE and HA [a] and HA [b]) and covariate, main effect, and interaction effect analysis of the variable influence (the participants’ gender, age, and education level and environmental parameters were adjusted in each model). (3) Based on the f-value, value of p, and ES ɳp^2^ of the repeated measures ANOVA, the influence of HA (a) and GE on the improvement effect of each dependent variable index and effect of HA (a) and HA (b) on each dependent variable were tested. The impact of the improvement of the indicators (for each model, the demographic and sociological variables, individual factors, and environmental parameters were adjusted, and the interaction effect between the variables was eliminated).

## Results

### Comparison of differences in pre-test and post-test of dependent variables

#### Blood pressure

**Figure 5:**
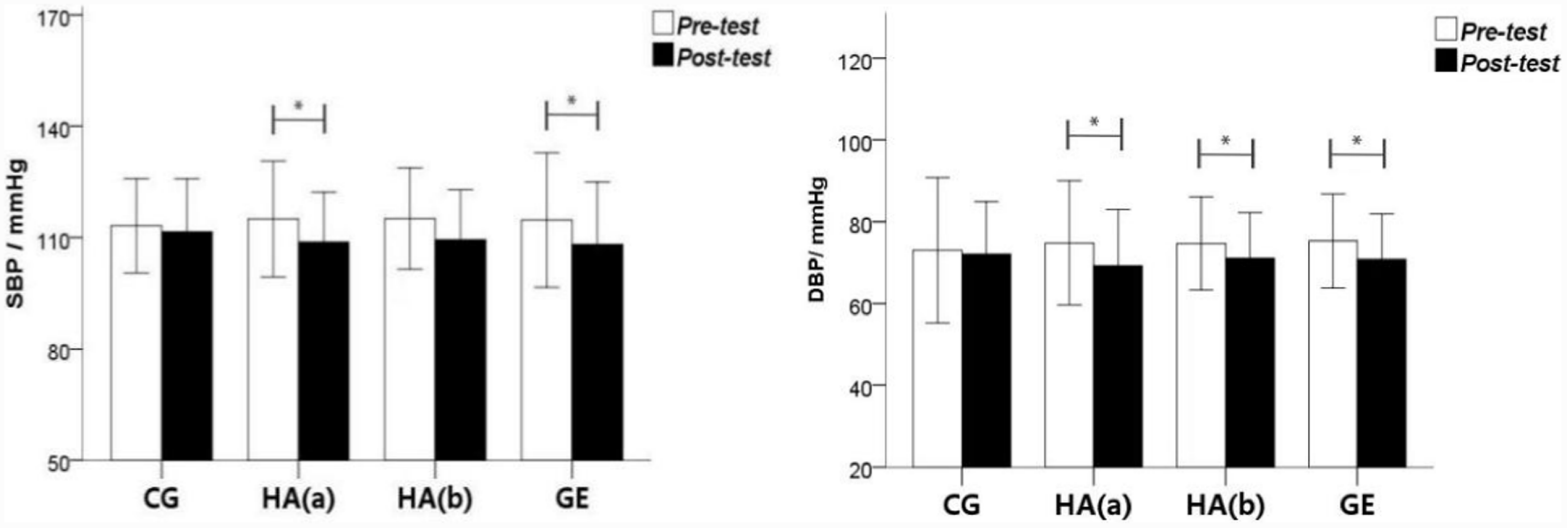
The trend of pre-test and post-test changes in SBP and DBP.

Table 1 shows no significant difference between the post-measured and pre-measured values of SBP and DBP in the sedentary control group. In the group with green plant horticultural activities, the SBP of the subjects decreased from 114.97 ± 7.81 mmHg to 108.79 ± 6.70 mmHg after the test, which showed a significant difference (*F* = 0.923; *p* = 0.049; ɳp^2^ = 0.126). The DBP of the subjects decreased from 74.82 ± 7.58 mmHg to 69.26 ± 6.85 mmHg on the post-test, showing a significant difference between the post-test and pre-test (*F* = 3.034; *p* = 0.024; ɳp^2^ = 0.322). In the non-green horticultural group, the DBP decreased from 74.71 ± 5.74 mmHg to 71.05 ± 5.59 mmHg, showing a significant difference between the post-DBP and the pre-DBP (*F* = 1.193; *p* = 0.036; ɳp^2^ = 0.188). In the green exercise group, the SBP of the subjects decreased from 114.68 ± 9.06 mmHg to 108.13 ± 8.42 mmHg in the post-test, showing a significant difference between the post-test and the pre-test (*F* = 1.643; *p* = 0.019; ɳp^2^ = 0.241). DBP decreased from 75.29 ± 5.69 mmHg to 70.79 ± 5.55 mmHg post-test, showing a significant difference between the post-test and post-test (*F* = 1.509; *p* = 0.037; ɳp^2^ = 0.090). Shown in Figure 5, the difference between SBP and DBP in pre-test and post test.

**Table 1:** Difference test results of BP indicators.

	**CG**		**HA(a)**		**HA(b)**		**GE**	
	M ± SD	Multi-Factor ANOVA	M ± SD	Multi-Factor ANOVA	M ± SD	Multi-Factor ANOVA	M ± SD	Multi-Factor ANOVA
***SBP [mmHg]***								
Pre-test	113.13 ± 6.36	*F* = 1.601 *p* = 0.180 ɳp^2^ = 0.237	114.97 ± 7.81	F = 0.923 *p* = 0.049* ɳp^2^ = 0.126	115.08 ± 6.82	*F* = 1.826 *p* = 0.126 ɳp^2^ = 0.261	114.68 ± 9.06	F = 1.643 *p* = 0.019* ɳp^2^ = 0.241
Post-test	111.53 ± 7.15		108.79 ± 6.70		109.39 ± 6.72		108.13 ± 8.42	
***DBP [mmHg]***								
Pre-test	73.03 **±** 8.87	*F* = 0.423 *p* = 0.858 ɳp^2^ = 0.076	74.82 ± 7.58	F = 3.034 *p* = 0.024* ɳp^2^ = 0.322	74.71 ± 5.74	F = 1.193 *p* = 0.036* ɳp^2^ = 0.188	75.29 ± 5.69	F = 1.509 *p* = 0.037* ɳp^2^ = 0.090
Post-test	72.08 ± 6.41		69.26 ± 6.85		71.05 ± 5.59		70.79 ± 5.55	

The above models are designed as follows: intercept + gender + age + education level + mean ambient temperature + mean ambient humidity + mean ambient noise. (*) indicates a significant difference between post-test and pre-test, where *: *p* < 0.05; **: *p* < 0.01; ***: *p* < 0.001. CG: Control group; HA(a):Horticultural activity group with green plants; HA (b):Horticultural activity group without green plants; GE:Green exercise group.

#### Positive/negative affects

**Figure 6:**
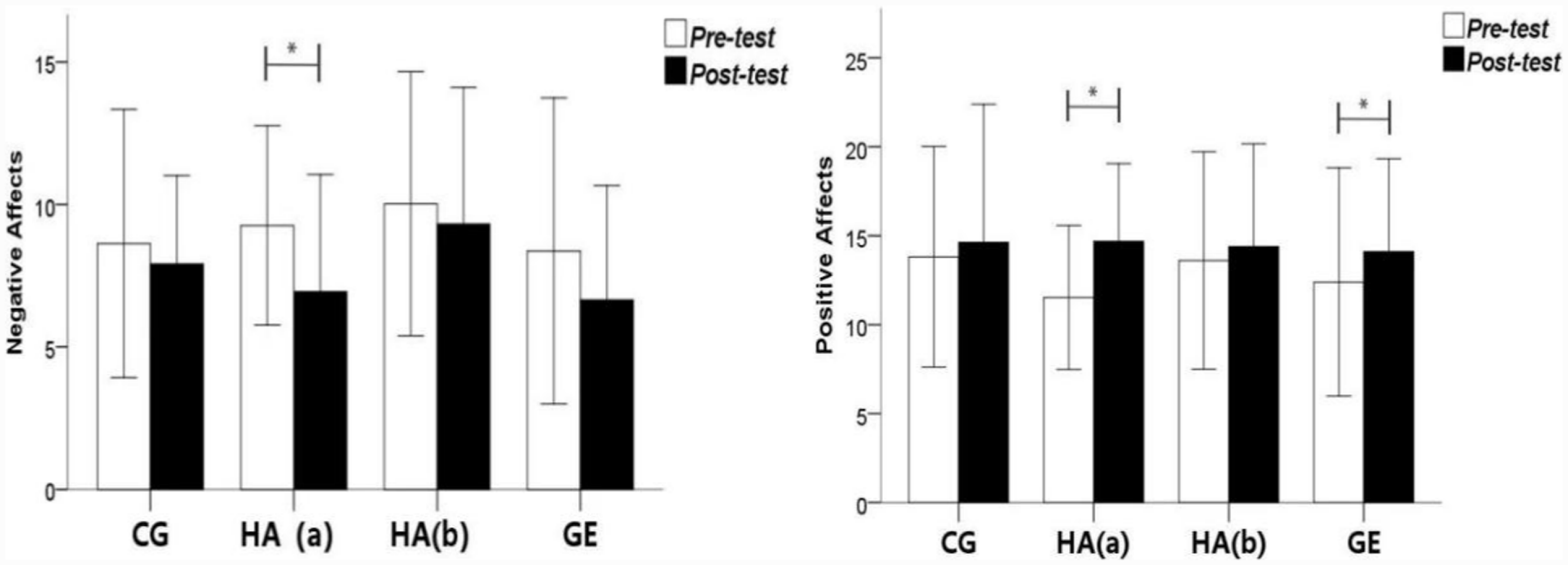
The pre-test and post-test change trends of negative affects and positive affects.

Table 2 shows that, in the sedentary control group, the negative and positive affects of the subjects showed no significant difference before and after the experiment. In the group with green plant horticultural activities, the negative affect score decreased from 9.26 ± 1.75 in the pre-test to 6.95 ± 2.05 in the post-test, showing a significant difference between the pre-test and post-test (*F* = 0.890; *p* = 0.050; ɳp^2^ = 0.122) and no significant difference in positive affect score. No significant difference in the negative and positive affects was found in the group without green horticultural activities in the pre and post-tests. In the green exercise group, the positive affect score of the subjects increased from 12.39 ± 2.31 in the pre-test to 14.11 ± 2.61 in the post-test, showing a significant difference between the pre-test and post-test (*F* = 2.903; *p* = 0.023; ɳp^2^ = 0.360). Shown in Figure 6, the difference between postive and negative affects in pre-test and post test.

**Table 2:** Difference test results of real-time affective indicators.

	**CG**		**HA(a)**		**HA(b)**		**GE**	
	M ± SD	Multi-Factor ANOVA	M ± SD	Multi-Factor ANOVA	M ± SD	Multi-Factor ANOVA	M ± SD	Multi-Factor ANOVA
***Positive Affects***								
Pre-test	6.61 **±** 2.35	*F* = 1.981 *p* = 0.099 ɳp^2^ = 0.277	9.26 ± 1.75	*F* = 0.890 *p* = 0.050* ɳp^2^ = 0.122	10.03 ± 2.31	F = 2.093 *p* = 0.083 ɳp^2^ = 0.288	8.37 ± 2.28	F = 2.049 *p* = 0.089 ɳp^2^ = 0.284
Post-test	5.72 ± 1.53		6.95 ± 2.05		9.32 ± 2.39		6.66 ± 2.01	
***Negative Affects***								
Pre-test	13.82 ± 3.10	*F* = 2.044 *p* = 0.089 ɳp^2^ = 0.283	11.53 ± 2.02	*F* = 1.223 *p* = 0.021* ɳp^2^ = 0.160	13.61 ± 3.05	*F* = 1.118 *p* = 0.375 ɳp^2^ = 0.178	12.39 ± 3.21	F = 2.903 *p* = 0.023* ɳp^2^ = 0.360
Post-test	14.63 ± 3.87		14.68 ± 2.18		14.39 ± 2.88		14.11 ± 2.61	

The above models are designed as follows: intercept + gender + age + education level + mean ambient temperature + mean ambient humidity + mean ambient noise. (*) indicates a significant difference between post-test and pre-test, where *: *p < 0.05*; **: *p < 0.01*; ***: *p < 0.001*. CG:Control group; HA(a):Horticultural activity group with green plants; HA (b):Horticultural activity group without green plants; GE:Green exercise group.

#### Heart rate variability

**Figure 7:**
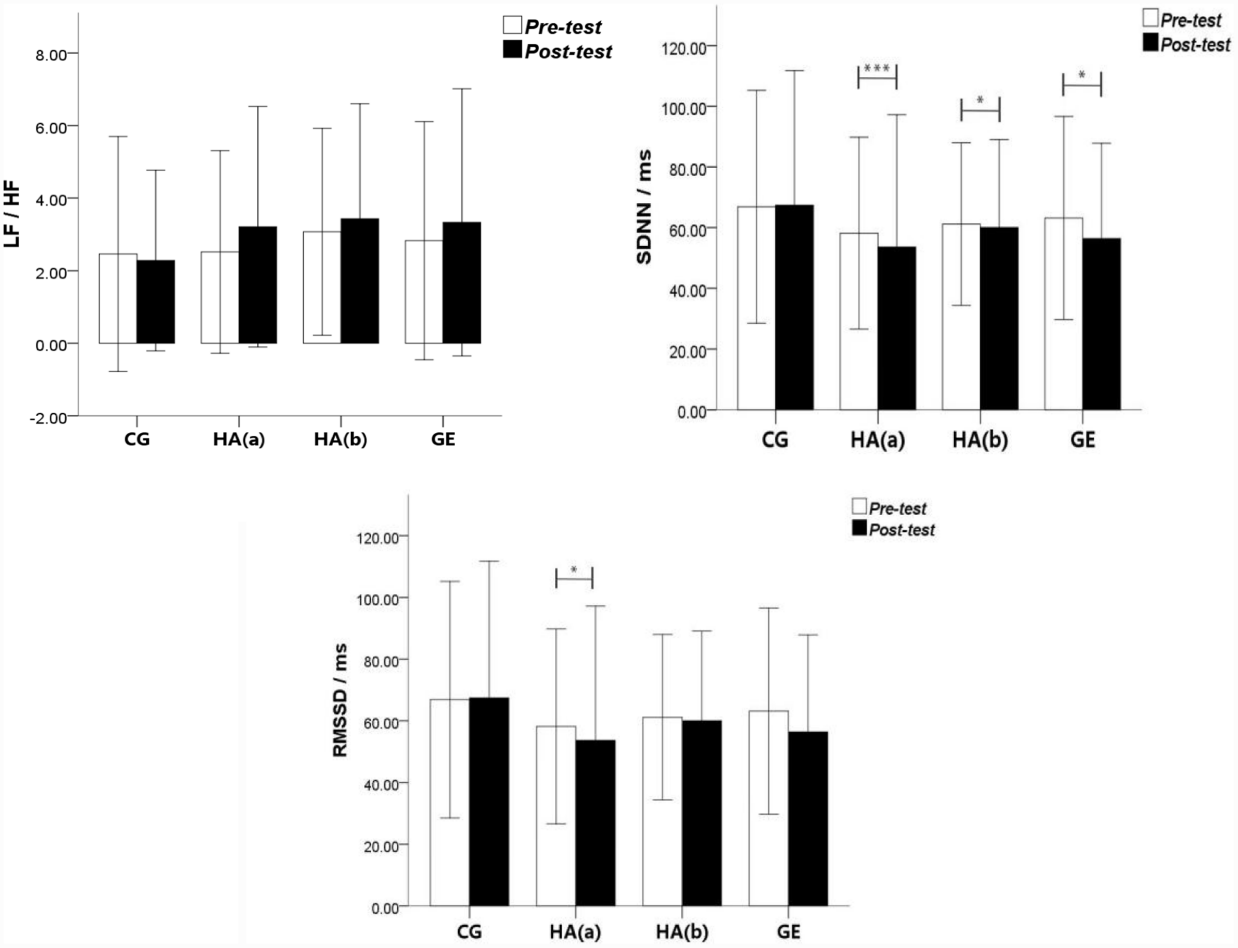
Pre-test and post-test change trends of HRV indexes LF/HF, SDNN and RMSSD.

Table 3 shows the improvement effect of HRV. In the sitting control group, no significant difference was found between the post-measured value and the pre-measured value of the three indicators of HRV. In the group with green plant horticultural activities, the SDNN index showed a decreasing trend from 58.17 ± 15.79 to 53.65 ± 21.77 and a significant difference between the latter and the former (*F* = 5.727; *p* = 0.001; ɳp^2^ = 0.526). RMSSD index value showed a specific decreasing trend from 40.19 ± 9.51 in the pre-test to 34.63 ± 10.81 in the post-test; the post-test value also showed a significant difference compared with the pre-test value (*F* = 3.030; *p* = 0.024; ɳp^2^ = 0.321). In the group without green plant horticultural activities, the LF/HF index and RMSSD post-test value showed no significant difference compared with the pre-test value, but SDNN showed a significant difference compared with the pre-test value (*F* = 2.860; *p* = 0.025; ɳp^2^ = 0.356). In the green exercise group, the SDNN index value showed a specific decreasing trend from 63.19 ± 16.73 in the pre-test to 56.41 ± 15.69 in the post-test. The post-test value showed a significant difference compared with the pretest value (*F* = 2.462; *p* = 0.046; ɳp^2^ = 0.323). Shown in Figure 7, the difference between HRV in pre-test and post test.

**Table 3:** The difference in test results of the heart rate variability.

	**CG**		**HA(a)**		**HA(b)**		**GE**	
	M ± SD	Multi-Factor ANOVA	M ± SD	Multi-Factor ANOVA	M ± SD	Multi-Factor ANOVA	M ± SD	Multi-Factor ANOVA
***LF/HF***								
Pre-test	2.46 ± 1.61	*F* = 1.840 *p* = 0.124 ɳp^2^ = 0.263	2.51 ± 1.39	*F* = 1.052 *p* = 0.412 ɳp^2^ = 0.169	3.07 ± 1.42	*F* = 1.486 *p* = 0.216 *ɳp^2^* = 0.223	2.82 ± 1.64	*F* = 1.771 *p* = 0.138 ɳp^2^ = 0.255
Post-test	2.28 ± 1.24		3.21 ± 1.65		3.43 ± 1.58		3.33 ± 1.84	
***SDNN [ms]***								
Pre-test	66.88 ± 19.18	*F* = 0.474 *p* = 0.822 ɳp^2^ = 0.084	58.17 ± 15.79	F = 5.727 *p* = 0.001*** ɳp^2^ = 0.526	61.16 ± 13.41	F = 2.860 *p* = 0.025* ɳp^2^ = 0.356	63.19 ± 16.73	F = 2.462 *p* = 0.046* ɳp^2^ = 0.323
Post-test	67.42 ± 22.17		53.65 ± 21.77		60.04 ± 14.51		56.41 ± 15.69	
***RMSSD [ms]***								
Pre-test	41.66 ± 22.01	*F* = 0.166 *p* = 0.984 ɳp^2^ = 0.031	40.19 ± 9.51	F = 3.030 *p* = 0.024* ɳp^2^ = 0.321	39.41 ± 12.43	*F* = 0.885 *p* = 0.518 ɳp^2^ = 0.146	40.52 ± 14.31	*F* = 1.873 *p* = 0.117 ɳp^2^ = 0.266
Post-test	37.41 ± 18.82		34.63 ± 10.81		37.47 ± 10.76		35.03 ± 13.77	

The above models are designed as follows: intercept + gender + age + education level + mean ambient temperature + mean ambient humidity + mean ambient noise. (*) indicates a significant difference between post-test and pre-test, where *: *p* < 0.05; **: *p* < 0.01; ***: *p* < 0.001. CG:Control group; HA(a):Horticultural activity group with green plants; HA (b):Horticultural activity group without green plants; GE:Green exercise group.

### Difference of dependent variables between green exercise group and horticultural group with green plants

The repeated measures ANOVA model in Table 4 showed no significant difference in the influence of the green exercise group and the horticultural group on the subjects’ SBP and DBP. As for the improvement effect of real-time affect, only the negative affect was significantly different between the green exercise group and the green horticultural group (*F* = 3.310; ɳp^2^ = 0.046; *p* = 0.037). Compared with green exercise (Table 2, *F* = 2.049; *p* = 0.089; ɳp^2^ = 0.284), the horticultural group with green plants was more conducive to improving negative affects (Table 2, *F* = 0.890; *p* = 0.050; ɳp^2^ = 0.122). However, no significant difference was found between the green exercise group and the horticultural group in the positive affects of the subjects. As for the improvement effect of HRV, the green exercise group and the green horticultural activity group did not significantly differ in SDNN and RMSSD time-domain indexes and did not achieve a significant difference in LF/HF frequency domain indexes.

**Table 4:** The effect of green exercise and horticultural activities on the variation of the dependent variable.

***Dependent Variable***	***GE* VS. *HA(a)***		
	***F***	***ɳp*** ^***2***^	***p***
*SBP*	0.056	0.001	0.813
*DBP*	0.566	0.08	0.455
*Positive Affects*	0.053	0.001	0.819
*Negative Affects*	3.310	0.046	0.037*
*LF/HF*	3.642	0.051	0.060
*SDNN*	0.274	0.004	0.621
*RMSSD*	0.254	0.004	0.616

Gender, age, education level, mean temperature, mean humidity, and mean noise were adjusted for the above models based on the repeated ANOVA test. * is the significant difference between GE and HA(a). [GE:Green exercise group; HA(a):Horticultural activity group with green plants].

### Difference comparison of dependent variables between The group with and without green plant horticultural activities

In the repeated measures ANOVA model, as shown in Table 5, the same horticultural activity pattern with or without green plants had no significant difference in the effects on the subjects’ SBP and DBP. For the improvement effect of real-time affect, the same horticultural activity pattern with or without green plants significantly differed in the negative affect score (*F* = 2.157; ɳp^2^ = 0.119; *p* = 0.031). Compared with the group without green plant activities (Table 2, *F* = 2.093; *p* = 0.083; ɳp^2^ = 0.288), the green plant activity group was more conducive to improving the negative affects of the subjects (Table 2; F = 0.890; *p* = 0.050; ɳp^2^ = 0.122). As for the improvement effect of HRV, the same horticultural activity mode with and without green plants had a significant difference in the effect of the SDNN time-domain indexes of the subjects (*F* = 1.053; ɳp^2^ = 0.015; *p* = 0.039). Compared with the group without green plant activities (Table 3; *F* = 2.860; *p* = 0.025; ɳp^2^ = 0.356), the green plant activity group was more conducive to the improvement of the SDNN index (Table 3, *F* = 5.727; *p* = 0.001; ɳp^2^ = 0.526). Furthermore, a significant difference was observed in the effect of the RMSSD time-domain index (*F* = 2.225; ɳp^2^ = 0.032; *p* = 0.014).

**Table 5:** The influence of the presence or absence of green plants in horticultural activities on the variation of the dependent variable.

***Dependent Variable***	***HA(a)* vs. *HA(b)***		
	***F***	***ɳp*** ^***2***^	***p***
*SBP*	0.327	0.005	0.569
*DBP*	2.667	0.038	0.107
*Positive Affects*	1.294	0.153	0.096
*Negative Affects*	2.157	0.119	0.031*
*LF/HF*	3.028	0.043	0.086
*SDNN*	1.053	0.015	0.039*
*RMSSD*	2.225	0.032	0.014*

Gender, age, education level, mean temperature, mean humidity, and mean noise were adjusted for the above models based on the repeated ANOVA test. * is the significant difference between HA(a) and HA(b). [HA(a):Horticultural activity group with green plants; HA (b):Horticultural activity group without green plants].

## Discussion

### Effects of horticulture activities and green exercise on affect improvement

The analysis showed that the two physical activity intervention groups significantly influenced the difference in the negative affect. Compared with the GE group, the green horticultural activity groups exerted a more substantial effect on negative affect improvement. The results revealed that the green horticultural activity groups also had an excellent promoting impact on negative affect improvement. Horticultural activities are believed to be able to achieve the activity effect of green exercise. After the subjective and objective evaluation of the interventions (including positive and negative affect and HRV), the horticultural activity groups showed that the improving effect of various aspects may have been caused by the intensity of the physical activity and greenery synergy, resulting from interaction and contact. The participants engaged in horticultural activities for pleasure, physical activity, and increased communication opportunities with others, which can reduce loneliness and significantly improve affect.

The experiment combined the subjective and objective evaluation indices to comprehensively evaluate the participants’ affective improvement before and after the intervention activities, which may complement each other, with solid objectivity and accuracy (Wooller et al., 2015; Rogerson et al., 2016b). Subjective evaluation methods for positive and negative affect were used and verified in many studies (Thompson and E, 2007; Liu et al., 2012). For example, Melissa et al. (2014) conducted a study on participants free walking in green spaces with unlimited intensity and time, compared the participants’ subjective affective evaluation before and after walking, and found that positive dynamic scores increased significantly, whereas negative affective scores decreased after walking (Melissa et al., 2014). John et al. (2018) developed an exercise in a simulated green space in a laboratory, compared the results of the pretest and post-subjective scales, and determined that green practice is conducive to improving affect (John et al., 2018).

With the same-intensity green horticultural activities and green exercise, the positive/negative affect scores of the subjective evaluation indices as well as the changes in the objective evaluation indices of SBP and DBP reflected that the participants’ negative affect improved to a certain extent before and after the intervention. In the examination of the HRV index, the RMSSD and SDNN values exhibited a downward trend in the posttest stage compared with those in the pretest stage, thereby reflecting the modulating effect of sympathetic activity, which predominated in the posttest intervention activity and increased the positive affect of the participants (Corrales et al., 2012; Rhie et al., 2017). The LF/HF values in the frequency domain showed an upward trend compared with those in the premeasurement stage. The results indicated that the autonomic nerve regulation balance was biased toward the sympathetic nerve, thereby allowing sympathetic modulation to dominate (Melissa et al., 2014). For the HRV index, the comprehensive evaluation of the frequency-domain analysis and time-domain analysis revealed that sympathetic neural activity played a leading role when the participants returned to the calm state after the intervention activity, which improved their positive affect to a certain extent (Beauchaine and Thayer, 2015; John et al., 2018).

### Comparative analysis of affect improvement effect of the same horticulture activity pattern with and without green plants

The analysis revealed that with and without green plants were involved in the same horticultural mode in testing the impact of negative affect. The horticultural activity group with green plants tried to exert a substantial effect on negative affect improvement. Simultaneously, in the two horticultural activity intervention groups, the change in the influence of the participants’ SDNN and RMSSD indices was also significant. The horticultural activity group with green plants tried to exert a substantial influence on the improvement of the HRV index, thereby showing that physical activity coordination with green plants involved considerable assistance to improve human affect. Thus, green plants can help in physical and mental health improvement. The critical factors showed that plants can positively impact individuals’ creative tasks. When one can directly see or touch green plants, his/her affect can be in a positive state. The study results showed that SBP and DBP decreased after horticultural activities and green exercise, which are associated with reduced sympathetic tone and peripheral resistance after exercise (Carpio-Rivera et al., 2016; Igarashi and Nogami, 2017). However, the exercise environment may also change this effect. Previous studies showed that BP increases significantly after walking in downtown areas, which may be related to noise and air pollution (Ahmad et al., 2018; Bridget, 2018). The results also demonstrated that exercising in a forest or urban park is beneficial for avoiding negative urban factors to promote health. The present study also found that compared with no such activities of the CG, green exercise and horticultural activities were more effective in reducing SBP and DBP. As changes in BP are regulated autonomically, they are consistent with the HRV index results (Mena-Martín et al., 2010). Other studies suggested that in addition to vision, chemicals released by flowers and trees may also be responsible for the predominant modulation of parasympathetic activity that can reduce BP, but the mechanism remains unclear (Freeman et al., 2013). Physical activity intervention is essential to preventing and treating hypertension (Silva et al., 2017; Zhu et al., 2021). According to the results of this study, the effects of green exercise and horticultural activities on cardiovascular activity can be considered as effective.

The horticultural activity’s effect on the improvement of the human affect is from a certain intensity under the impact of the physical activity or a physical activity combined with the green function of synergy effect. The process of horticultural intervention in the groups without green plants, with green plants, and control can provide sufficient evidence for studying the effect of horticultural activities to improve affect. Previous studies have suggested that the benefits of green activities may be due to an individual’s overall perception of objects in a natural environment rather than simply being green (Dravigne et al., 2008). In research discovery, the green plant can help effectively recover mental workers’ physiological conditions. Suppose it reduces visual fatigue and alleviates working pressure. In that case, the green plant can create a relatively relaxed and cheerful bedroom environment atmosphere, which is beneficial to the health of a person’s body and mind (Bringslimark, 2007). At the same time, plants positively impact people to complete creative tasks. When people can directly see green plants, their affect can be more positive, and their study and work efficiency will significantly improve. Compared with the pictures of an indoor environment without plants, when people watched the images of an indoor environment with green plants, their tension and anxiety decreased, and their affects were more stable, indicating that the office environment with green plants had a more positive effect on people’s psychology. People are happier when surrounded by greenery (Chen and Ping, 2005; Dravigne et al., 2008). According to the results of this study for the same model of horticultural groups with green plants and no green plants, the two groups of participants in the subjective evaluation showed positive and negative affective scores, respectively, and the objective evaluation index reflected the changes of SBP and DBP degree. Horticultural intervention were negative affects after specific affect improvement was obtained. At the same time, the frequency domain index LF/HF increased, and the time domain index RMSSD and SDNN decreased, which reflected the improvement effect of positive affect (Schwerdtfeger and Gerteis, 2014; Zijlema et al., 2018).

### Limitations

1. As the participants of this study were all college students at Zhejiang Normal University, the results may not be easily extended to other populations. Furthermore, the overall cognitive level of the college students may be higher than that of other groups, which may affect the research results. Previous studies also conducted qualitative analyses on green exercise and horticultural activities for individuals with psychological problems. The empirical results showed that green exercise and horticultural activities under a certain activity intensity positively improved mental health. Meanwhile, studies on “green therapy” and “horticultural therapy” for improving mental health boomed in recent years. However, this experimental study did not compare the participants’ mental health, which should be the focus of future research on this group.
2. Improvement of living standards may lead to bias toward urbanization, children’s living environment, and science and technology. The elderly have considerable opportunities to interact with the natural environment and green plants, and different people have different levels of affective closeness to the natural environment. Exercise activities in the natural environment also require further examination. Green exercise and horticultural activities may have other physical and mental health benefits for other groups of people. However, this study used no specific quantitative standards for defining green naturalness and green plant quality. Thus, measuring the naturalness of the physical activity environment objectively is necessary. Moreover, this study provided no clear operational definition of green exercise and horticultural activities of the same mode with or without green plants. The test environment and conditions with or without green plants were chosen through comprehensive comparison based on previous studies. Thus, the quality of the natural space environment, variety and quality of plants, and specific horticultural activity operational mode should be ensured in future research to maximize the benefits of green exercise and horticultural activities.

### Advantages

1. The literature retrieval and sorting results revealed that many experimental studies comparing the effects of horticultural activities and other physical activities on affect improvement did not effectively consider the intensity of the horticultural activities. A possible reason for this shortcoming is that the intensity assessment of horticultural activities is in its infancy, and no mature means and methods similar to the intensity assessment of green exercise are available. Thus, the horticultural activities of the experiment groups should be determined. In addition, for the GE group, the physical activity intensity and activity time should be compared with the physical activity intensity and activity time of the horticultural activity groups as much as possible to avoid experiment errors owing to the problem of considerable physical activity intensity and time differences, which may affect the results of the dependent variable indices.
2. Does the improvement effect of horticultural activities on human affect derive from physical activity under a certain intensity or from the synergistic effect of the physical activity and green plants? Horticultural activities with the same activity intensity and operational mode with and without green plants can provide substantial evidence for the examination of the effect of horticultural activities on affect improvement.

## Conclusion

1. The equivalent strength of horticultural and green exercise group’s affective and physiological parameters reflect the SBP, DBP, and HRV index (SDNN, RMSSD, and LF/HF), and subjective evaluation indexes show no significant difference between positive affects. Negative affects only had personal evaluation as an indicator. The horticultural group with green plants was slightly better than the green exercise group. The same intensity of horticultural activities with green plants is believed to have the ability to achieve the improvement effect of green exercise on affect. Thus, people can choose green exercise or horticultural activities according to their preferences and physical characteristics.
2. Under the same horticultural mode, the physiological parameters reflect the SDNN, RMSSD, and negative affective feelings, and the improvement effect of the subjective evaluation indices of the horticultural activity group with green plants is superior to that of the horticultural activity group with no green plants. The dynamic and physiological parameters reflect the SBP, DBP, and positive affect, and the LF/HF of the two groups exhibits no significant differences. Therefore, horticultural activities with green plants have a better effect on affect improvement than horticultural activities without green plants under the same horticultural pattern. Thus, individuals can choose and interact with different plant types according to their preference during horticultural activities to achieve other activity effects.

## Data availability statement

The raw data supporting the conclusions of this article will be made available by the authors, without undue reservation.

## Ethics statement

The studies involving human participants were reviewed and approved by The ethical approval document authorized by the Academic Research Ethics Committee of Zhejiang Normal University (No. ZSDD2021011). The patients/participants provided their written informed consent to participate in this study.

## Author contributions

XH made a substantial contribution to the concept and design, acquisition of data or analysis, and interpretation of data. MT made a substantial contribution to acquisition of data or analysis, and interpretation of data, and wrote the first draft of paper. LL and JG made a substantial contribution to acquisition of data and part contribution to acquisition analysis. All authors contributed to the article and approved the submitted version.

## Funding

This study was supported by the Social Science Planning Project of Zhejiang Province, China (Project No. 22JCXK01Z).

## Conflict of interest

The authors declare that the research was conducted in the absence of any commercial or financial relationships that could be construed as a potential conflict of interest.

## Publisher’s note

All claims expressed in this article are solely those of the authors and do not necessarily represent those of their affiliated organizations, or those of the publisher, the editors and the reviewers. Any product that may be evaluated in this article, or claim that may be made by its manufacturer, is not guaranteed or endorsed by the publisher.
